# SBR operational strategies for directing mixed microbial cultures toward levulinic acid production using hexoses

**DOI:** 10.3389/fbioe.2026.1726739

**Published:** 2026-06-01

**Authors:** F. Pinto-Ibieta, F. Cabrera, P. Díaz, J. A. Martínez-Ruano, A. Serrano

**Affiliations:** 1 Departamento de Procesos Industriales, Facultad de Ingeniería, Universidad Católica de Temuco, Temuco, Chile; 2 Departamento de Ciencias Veterinarias y Salud Pública, Universidad Católica de Temuco, Temuco, Chile; 3 Departamento de Ingeniería Química, Facultad de Ingeniería y Ciencias, Universidad de La Frontera, Temuco, Chile; 4 Departamento de Microbiología, Facultad de Farmacia, Universidad de Granada, Campus de Cartuja S/n, Granada, Spain; 5 Instituto de Investigación del Agua, Universidad de Granada, Granada, Spain

**Keywords:** aeration rate and OLR optimization, beet molasses valorization, feast and famine (F/F) culture strategy, levulinic acid biological production, mixed microbial cultures (MMCs)

## Abstract

To date, levulinic acid production by mixed microbial cultures operated under feast and famine regimes has been reported only using mixtures of hexoses and pentoses derived from lignocellulosic hydrolysates. This research presents the first report on the production of LA using a synthetic hexose mixture that mimics the sugar composition of pre-treated beet molasses as the main substrate. Molasses, characterized by its high sugar content and low lignin levels, requires only mild pretreatment, thereby facilitating hexose extraction and minimizing the formation of inhibitors such as hydroxymethylfurfural (HMF). Optimization of molasses pretreatment, combined with adjustments to aeration rate and organic loading rate (OLR), revealed that a moderate aeration rate (3 L/min) and an OLR of 3 g COD/L were optimal for LA production, achieving yields of up to 27.6% ± 1.1% (g LA/g VS). Lower aeration rates (<3 L/min) limited oxygen availability, thereby restricting microbial activity and LA synthesis, whereas higher aeration rates (>3 L/min) may induce oxidative stress, diverting energy from LA production to cellular maintenance. Similarly, high OLR promoted biomass growth, thereby competing with LA production for resources and reducing overall yields. These findings underscore the importance of controlling aeration rate and OLR to optimize LA yields and demonstrate the potential of glucose and fructose derived from hydrolyzed molasses to valorize sugar by-products.

## 1 Introduction

Beet molasses from the sugar industry is typically generated at 4%–6% of the processed sugar beet mass and could be a valuable resource for biotechnological and industrial processes (Bruni and Terrell, 2022; Camacho et al., 2022). Its high sucrose content (70% w/w) as a glucose dimer, together with the presence of amino acids, organic acids, and minerals, enhances its potential as a substrate for diverse bio-applications (Karaman et al., 2017; Jamir et al., 2021). Unlike lignocellulosic residues such as sugarcane bagasse or grape pomace, molasses lacks recalcitrant lignocellulosic fibers (Schmetz et al., 2019; Palmonari et al., 2020). Consequently, pretreatment mainly involves sucrose hydrolysis to glucose and fructose rather than lignin depolymerization, resulting in milder processing conditions, lower energy requirements, and reduced formation of inhibitory furans (Nie et al., 2013; Mustafa et al., 2023). Beet molasses can undergo Maillard reactions due to its protein content. These reactions may lead to the formation of inhibitors such as hydroxymethylfurfural (HMF) and furfural (Wang et al., 2022). Therefore, regulating the hydrolysis conditions of molasses is crucial to ensuring the quality of sugars after hydrolysis, preventing them from adversely affecting subsequent processes (Yang et al., 2022; Mayasri, 2023; Sadarman et al., 2023).

Hydrolyzed beet molasses currently serves as a valuable substrate for producing biofuels, carbohydrate derivatives (such as pullulan, dextran, and fructo-oligosaccharides), enzymes, and various organic acids (Jeong et al., 2018; Zhang et al., 2021; Mustafa et al., 2023). Among these, levulinic acid (LA) holds significant potential due to its applications across industries, as it is a precursor for pharmaceuticals, agrochemicals, flavors, fragrances, personal care products, resins, coatings, plasticizers, and fuel additives (Ukawa-Sato et al., 2023). LA is commonly produced from lignocellulosic biomass under chemical catalysis (Habe et al., 2020; Riveiro et al., 2020). LA production has also been studied in pure cultures, where host selection and substrate composition played a decisive role in LA biosynthesis (Vila-Santa et al., 2021; Kim et al., 2024). As an alternative, production using mixed microbial cultures (MMCs) would offer key advantages over pure cultures, including lower production costs and the ability to utilize residual raw materials like hydrolyzed molasses (Mohamad Fauzi et al., 2019; Fang et al., 2020).

The production of value-added compounds from MMCs has been widely studied for polyhydroxyalkanoates (PHA) production using a controlled feed setup in sequential batch reactors (SBR) through feast and famine (F/F) culture regime (Yin et al., 2019; Marchetti et al., 2022; Diankristanti et al., 2024). Using F/F culture regimes favors the selection of microorganisms capable of metabolizing the substrate, discarding non-specialized ones, and creating selective pressure that enhances metabolic adaptation to nutrient variability, thus promoting PHA production through nutrient abundance and limitation cycles (Fu et al., 2023; Morya et al., 2023). Beyond PHA, other value-added compounds by MMC could be produced using F/F culture strategy, such as glycogen, astaxanthin, polyphosphates, polyglucose (PG), triglycerides (TGA), adipic acid (AA), and/or LA (Pinto-Ibieta et al., 2021; Katagi et al., 2024; Marchetti et al., 2024). In fact, different authors have reported that MMCs could be driven to produce a target compound by managing key SBR operational conditions, such as the concentration of dissolved oxygen (DO), the organic loading rate (OLR), and the substrate composition (Pinto-Ibieta et al., 2021; Katagi et al., 2024). Previous research reported that, for LA production using hexose-pentose mixtures from hydrolyzed grape pomace as a carbon source, an aeration rate of 3 L/min yielded the highest LA production and yield. In comparison, higher aeration rates (5 L/min) and lower aeration rates (1 L/min) may be insufficient, decreasing the LA yield (Correa-Galetote et al., 2024). This aligns with reports on LA production from hemicellulose hydrolysate rich in xylose; higher LA production has been correlated with lower DO during operation, suggesting that LA production can be stimulated at DO levels below 3 mg/L (Pinto-Ibieta et al., 2021). Likewise, high OLR (<3 g COD/(L·day)) has also been correlated to an increased LA production, while lower OLR values (<2.5 g COD/(L·day)) result in reduced LA production and yields (Pinto-Ibieta et al., 2021; 2023; Correa-Galetote et al., 2024).

These differences in LA yields and production may also be influenced by substrate composition, beyond the optimal operating variables. Notably, the studies reviewed have used substrates composed of both hexoses and pentoses, primarily glucose and xylose. For example, glucose-rich substrates (64% glucose and 31% xylose) promote biomass production, leading to higher yields, such as 2.7 g LA/L with 35% LA production (Correa-Galetote et al., 2024). In contrast, substrates with a higher proportion of xylose, such as 79.7% xylose and 2.3% glucose, tend to accumulate more LA directly, resulting in 0.9 g LA/(L·day) with 37% production (Pinto-Ibieta et al., 2020). However, substrates composed exclusively of hexoses, such as glucose and fructose, have not yet been explored, leaving potential impacts on LA production and yield under investigation. Despite these general trends, it is necessary to assess the suitability of the molasses hydrolysate, enriched in glucose and fructose, as a substrate for LA production, and to examine how the production process is affected by the main operational parameters.

Therefore, this study evaluates the feasibility of producing LA biologically in an SBR operating under a feast and famine regime, using hexoses (fructose and glucose) as the main carbon source for MMC. Firstly, the hydrolysis pre-treatment of molasses was optimized to maximize the conversion of sucrose into hexoses (glucose and fructose) by varying the hydrolysis conditions: acid, time, and temperature. After that, obtaining LA from hydrolyzed molasses was subsequently optimized by evaluating the impact of varying (1) the aeration rate and (2) the OLR. To the best of our knowledge, this is the first report describing LA production from a synthetic hexose mixture designed to simulate the sugar profile of hydrolyzed beet molasses, expanding the possibilities for sugar-industry residue valorization.

## 2 Materials and methods

### 2.1 Pre-treatment: acid hydrolysis of beet molasses

Beet molasses obtained from Industria Azucarera Nacional S.A. (IANSA, Chillán, Ñuble Region, Chile) was used as substrate and stored at −20 °C prior to acid hydrolysis to preserve its physicochemical characteristics. The physicochemical composition of the beet molasses used in this study is presented in the Supplementary Table S1. Acid pretreatment of beet molasses for hexoses production was optimized by varying temperature (60, 75, and 90 °C), sulfuric acid concentration (1, 5.5, and 10% v/v), and reaction time (30, 75, and 120 min). A 3-factor, 3-level response surface design of experiments with three central points was employed to determine the optimal conditions (Supplementary Table S2). The experiments were carried out in 5 mL bath reactors at 100 rpm, with a molasses concentration of 50 g/L and temperature controlled by a thermoregulated bath. At the end of each test, liquid samples were collected from the reactors, centrifuged at 6000 rpm for 10 min, and subsequently filtered through polyvinylidene fluoride (PVDF) filters with a pore size of 0.22 µm for the analysis of hexoses (glucose and fructose), HMF and furfural, as described in Section 2.4.

### 2.2 SBR configuration and operation

Two experimental stages were conducted using sequential batch reactors (SBRs) to evaluate operational conditions that may influence levulinic acid production by mixed microbial cultures (MMC). In the first stage, the effect of aeration rate was assessed using 12 SBRs, with four aeration conditions evaluated in triplicate. In the second stage, 9 SBRs were operated to investigate the effect of organic loading rate (OLR), evaluating three OLR conditions in triplicate. The experimental conditions evaluated in both stages are summarized in Table 1. Each SBR consisted of a 1 L glass bottle with a working volume of 0.8 L. Aerobic sludge used as inoculum was obtained from the recirculation stream of an aerobic wastewater treatment reactor operated by Aguas Araucanía (Temuco, La Araucanía Region, Chile). A total of 10 L of sludge was collected and allowed to settle for 12 h under ambient conditions, yielding approximately 1.5 L of concentrated sludge that was used to inoculate the SBRs.

**TABLE 1:** Operational conditions evaluated in the SBR experiments during the two experimental stages.

Stage	Operational parameter evaluated	Aeration rate (L/min)	OLR (g COD/Lday)	C/N ratio
Stage I	Aeration rate	0.5, 1.0, 3.0, 5.0	2.0	20
Stage II	Organic loading rate (OLR)	3.0	2.0, 3.0, 4.0	20

Each experimental condition was performed in triplicate using independent SBRs.

Abbreviations: COD, chemical oxygen demand; OLR, organic loading rate; C/N ratio, carbon nitrogen ratio; SBR, sequential batch reactor.

The MMC enrichment process employed a feast and famine (F/F) culture strategy under aerobic dynamic feeding (ADF) conditions. Each cycle lasted 1,440 min (24 h) and was divided into three phases: a feeding phase (10 min), an aerobic reaction phase (1,420 min), and a removal phase (10 min). During the initial part of the aerobic reaction phase, the added substrate was rapidly consumed, corresponding to the feast phase, followed by a famine phase characterized by substrate depletion. The approximate duration of the feast (≈180–960 min) and famine phases (≈460–1,240 min) was estimated from the dissolved oxygen profiles under the different aeration conditions (Supplementary Figure S3). No settling phase was included, ensuring that all excess biomass was removed together with mixed liquor. Under these conditions, the sludge retention time (SRT) was equal to the hydraulic retention time (HRT), both corresponding to 4 days.

The pseudo-stationary state, defined as the point at which cycle parameters remained constant for at least five consecutive cycles, was used as an indicator of successful enrichment. Each F/F cycle began with the addition of 200 mL of a synthetic hexose mixture that mimicked the composition of sugars in the pre-treated beet molasses from the previous stage, containing 1.6 g COD/L of fructose and 1.4 g COD/L of glucose, corresponding to a total organic loading rate (OLR) of 3 g COD/(L·day), considering the 24 h cycle. The synthetic mixture was enriched with a mineral culture medium (Supplementary Material S5). The synthetic hexose mixture enabled a controlled, reproducible substrate composition during SBR enrichment and operation, thereby avoiding variability associated with the complex composition of real molasses hydrolysates.

The SBRs were inoculated with 3.6 g VS/L of aerobic sludge and operated at 28 °C, 60 rpm, and a C/N ratio of 20 within a pH range of 5.8–6.2. The pH was monitored throughout reactor operation but was not actively controlled. Each SBR was inoculated with approximately 600 mL of aerobic sludge and operated for 45 days during each experimental stage, and its operation was automated using a peristaltic pump linked to a compact DAQ system (cDAQ-9178 chassis, National Instruments, Austin, TX, United States) controlled via LabVIEW (National Instruments), as described by Pinto-Ibieta et al. (2023). Samples were collected in triplicate at 2–3 cycle intervals to monitor the evolution of levulinic acid (LA) production, biomass concentration, and carbon source uptake (glucose and fructose). Samples were stored at −20 °C before analysis.

### 2.3 Levulinic acid production using a synthetic hexose mixture in SBRs

To maximize levulinic acid (LA) production, aeration rate and organic loading rate (OLR) were sequentially evaluated in two experimental stages (Table 1). The experiments were conducted using the MMC enriched from aerobic sludge during the SBR operation described in Section 2.2. In the first stage, four aeration rate conditions (0.5, 1.0, 3.0, and 5.0 L/min) were evaluated in triplicate SBRs at a constant OLR of 2 g COD/(L·day). LA production was monitored throughout reactor operation, and the aeration rate yielding the highest LA yield was selected as the optimal condition for the subsequent stage. In the second stage, LA production was optimized by varying the OLR while maintaining the selected aeration rate. Three OLR conditions (2, 3, and 4 g COD/(L·day)) were evaluated in triplicate SBRs for 45 days. The OLR was modified by adjusting the concentration of the synthetic hexose mixture in the feeding solution while maintaining a constant influent volume per cycle (200 mL), corresponding to an HRT of 4 days. The composition of the mineral medium was adjusted to maintain a C/N ratio of 20 across the evaluated conditions. The reactors were inoculated with the MMC enriched in the SBRs that showed the highest LA production during the first stage. LA yields and substrate consumption were quantified to determine the effect of OLR on LA production. The levels and variables evaluated were selected based on previous studies (Fang et al., 2019; Pinto-Ibieta et al., 2021; Pinto-Ibieta et al., 2023; Correa-Galetote et al., 2024). The influence of dissolved oxygen (DO) during the process was evaluated indirectly by varying the applied aeration rate. Although DO was not used as a controlled operational parameter, DO concentration was monitored in the reactors to support the interpretation of the results obtained under different aeration conditions (Supplementary Figure S3).

### 2.4 Analytical methods

Substrate (hexoses) consumption was determined by measuring the concentration of reducing sugars in filtered samples using a 0.22 μm pore size PVDF membrane (Merck). Reducing sugars were quantified using the dinitrosalicylic acid (DNS) method, measuring absorbance at 540 nm using a GENESYS 10s spectrophotometer (Thermo Scientific, United States), with glucose (Merck, Germany) as the standard (Yin et al., 2019). Biomass was monitored through volatile solids (VS) and soluble chemical oxygen demand (sCOD), as determined by standard methods (AWWA, 2005). Dissolved oxygen (DO) concentration in the SBRs was monitored using a wireless optical dissolved oxygen sensor (PS 3246, PASCO Scientific, United States).

Levulinic acid (LA) was quantified according to Lappalainen and Dong (2019) and Serafim et al. (2024), after a washing step to avoid interference from dissolved sugars. The biomass was washed three times by centrifugation (12,000 g, 10 min), resuspended in distilled water, and lyophilized. The dried biomass was resuspended in 1 mL of acidified methanol (20% H_2_SO_4_) containing 0.65 mg/mL benzoic acid (internal standard, Sigma-Aldrich), then 1 mL of chloroform was added. Samples were incubated at 100 °C for 3.5 h in a thermoblock, cooled, and extracted with 0.5 mL of water. The chloroform phase was recovered, dried with molecular sieves (0.3 nm), and injected into a GC/MS system (Clarus 600, PerkinElmer) equipped with an ELITE 1701 column (30 m × 0.25 mm × 0.25 µm, PerkinElmer). Calibration curves were prepared by processing LA standards (Sigma-Aldrich) under identical conditions.

Moisture content of beet molasses was determined gravimetrically after drying in a convection oven at 105 °C for 2 h. Sugars (sucrose, glucose, fructose, xylose, arabinose) and degradation products (furfural, HMF) were analyzed by HPLC (YL9100 TechnoLab System) equipped with a Bio-Rad Aminex HPX-87H column (300 × 7.8 mm), using a refractive index detector (RID) for sugars and a diode array detector (DAD) for furfural and HMF. The mobile phase consisted of 5 mM H_2_SO_4_ at a flow rate of 0.6 mL/min, with the column maintained at 65 °C. Samples were diluted in deionized water, treated with Carrez I/II, adjusted to pH 7.5–8.0 (NaOH 1 M), centrifuged, and filtered through 0.22 μm membranes before injection, following Serrano et al. (2023).

### 2.5 Data and statistical analysis

The acid hydrolysis of beet molasses (Section 3.1) was optimized using Design-Expert v13 through response surface methodology (RSM) to evaluate the effects of temperature, acid concentration, and reaction time (Supplementary Materials, S6). Statistical analyses for Sections 3.2, 3.3 were performed using SigmaPlot v15, in which differences among variables were first assessed using analysis of variance (ANOVA). When significant effects were detected (p < 0.05), mean values were compared using Tukey’s honestly significant difference (HSD) test at a 5% significance level. All measurements were conducted in triplicate, and results are presented as mean ± standard deviation (see Supplementary Materials S7 for more details).

## 3 Results and discussion

### 3.1 Optimization of the acid hydrolysis of molasses

**FIGURE 1:**
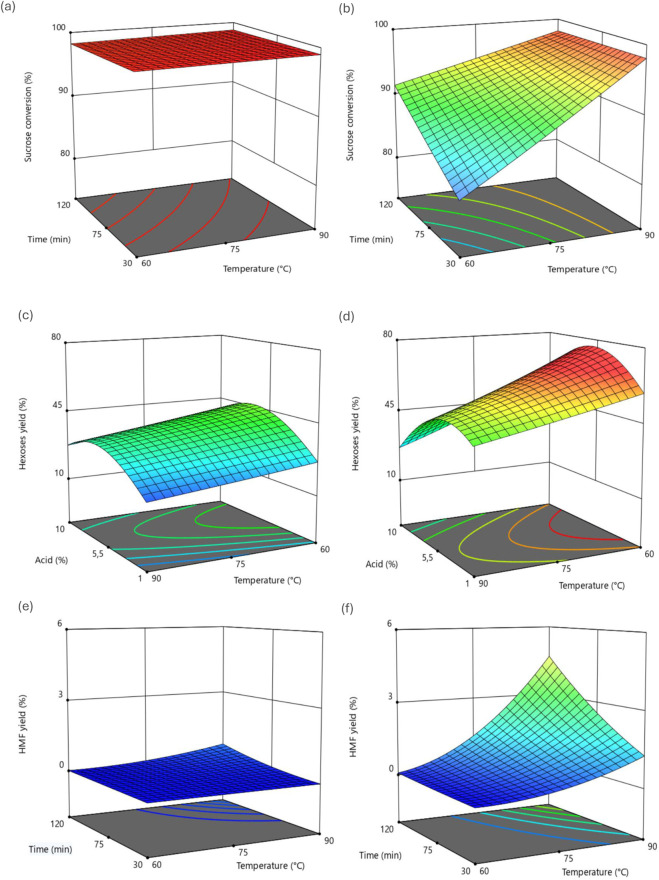
Response-surface (3D) plots of acid hydrolysis of sugar molasses. Sucrose conversion as a function of temperature (60°C–90 °C) and time (30–120 min) at **(a)** 10% v/v and **(b)** 1% v/v H_2_SO_4_. Hexose yield (glucose + fructose) versus temperature and time at **(c)** 30min and **(d)** 120min. HMF yield vs. temperature and time at **(e)** 1% v/v and **(f)** 10% v/v H_2_SO_4_.

Acid hydrolysis of beet molasses composition was evaluated at different temperatures, reaction times, and acid concentrations to determine sucrose conversion, hexose yield, and HMF yield (Figure 1). When acid concentration was maintained at 10% v/v of H_2_SO_4_, sucrose conversion remained above 90%, regardless of the reaction time (30–120 min) and temperature (60 °C–90 °C) (Figure 1a). In contrast, when the acid concentration was reduced to 1% v/v of H_2_SO_4_, lower temperatures and shorter reaction times significantly hindered sucrose degradation (Figure 1b). For instance, at 60 °C and a reaction time of 30 min, the sucrose conversion was 81% ± 1% w/w. Conversely, increasing the temperature to 90 °C resulted in a 98% ± 1% w/w conversion of sucrose, regardless of reaction time.

This suggests that under the most severe conditions evaluated (10% v/v H_2_SO_4_), almost all soluble sucrose was degraded into simpler compounds. This behavior does not correlate with an increased hexose yield (the combined yield of glucose and fructose). As shown in Figures 1c,d, the highest hexose yield was obtained with 5.5% v/v H_2_SO_4_, regardless of temperature. The best results were obtained at 60 °C with 5.5% v/v H_2_SO_4_ and a reaction time of 120 min, yielding a hexose yield of up to 70% w/w. Considering that the main component of beet molasses is sucrose (52.3% w/w), these results are consistent with those reported by Bower et al. (2008), who observed a reduction in the yield of hexoses (glucose and fructose) from the hydrolysis of sucrose under severe conditions (200 °C, 2.0% v/v H_2_SO_4_, 8 min) compared to milder conditions (160 C, 0.1 %v/v H_2_SO_4_, 12 min). Specifically, the yield in the severe conditions was only 10%, whereas under milder conditions it reached 75%.

The differences in sucrose conversion and hexose yield may also be due to the generation of undesirable compounds, such as HMF, under more severe pre-treatment conditions (Yang et al., 2022; Sadarman et al., 2023). Notably, no HMF generation was observed at a low acid concentration (1%), regardless of temperature or reaction time (Figure 1f). However, HMF production increased with temperature and reaction time when 10% v/v H_2_SO_4_ was used. The highest HMF formation was observed under the most severe conditions evaluated (10% v/v H_2_SO_4_, 90 °C, and 120 min), reaching 5.1% ± 1.0% w/w (Figure 1e).

Considering that the molasses studied contain approximately 8.7% crude protein (Supplementary Table S1), and that severe treatment conditions are known to promote sugar degradation (Bower et al., 2008), the observed disparity between mild and severe conditions regarding HMF generation could be attributed to Maillard type reactions between amino acids and reducing sugars (especially fructose), resulting in the formation of byproducts such as furfural, HMF, acetic acid, and formic acid (Yang et al., 2022; Mayasri, 2023; Sadarman et al., 2023). These reactions are catalyzed at higher temperatures and reaction times, which enhances the dehydration and conversion kinetics of sugars into HMF (Figure 1e) (Fan et al., 2011; Körner et al., 2019). So, the results indicate that although higher acid concentrations accelerate sucrose conversion, they must be carefully balanced with temperature and time to avoid hexose degradation and excessive HMF production. Nevertheless, the maximum HMF concentration achieved during molasses hydrolysis (0.05 g/L) is significantly below the 2.0 g/L threshold for furfural and HMF, which has been cited as the concentration that fully inhibits microbial activity in anaerobic digestion systems (Ghasimi et al., 2016).

Despite the HMF generation under the most severe pretreatment conditions, the maximum hexose yield obtained (26.5 ± 3 g/L) was consistent with values reported in the literature for similar sugar-rich substrates. Serrano et al. (2023) obtained 30 ± 1 g/L of simple sugar during the hydrolysis of grape pomace, although at more severe conditions (95 °C and 15% v/v H_2_SO_4_). Rodríguez-Gutiérrez et al. (2019) reported the production of 65 g/kg of simple sugars from thermally treated strawberry extrudate pretreated with 0.5% glacial acetic acid at 90 °C for 90 min. However, our results showed that low-temperature pretreatment was more effective for beet molasses, achieving a maximum hexose concentration of 45 ± 2 g/L. This can be explained by the high sucrose concentration in molasses and the absence of recalcitrant lignocellulosic fibers (Palmonari et al., 2020). This allows for efficient conversion of sucrose under milder conditions. Given the initial molasses concentration used in the experiments (50 g/L), this value corresponds to a hexose yield above 90%, consistent with the conversion results previously discussed. For comparison, the 60 °C temperature used in this study is 30 °C–35 °C lower than that used for grape pomace and strawberry.

Given that the evaluated variables lead to significant differences in sucrose conversion, hexose, and HMF yields, a mathematical model was applied to determine the optimal pretreatment conditions based on the interrelationships among the experimental factors (see Supplementary Materials S6 for more details). The fitted models are shown in Equations 1–3, where A, B, and C denote acid concentration, temperature, and reaction time, respectively:

$$\text{Sucrose conversion }\left(\%\right)=95.67+3.31A+3.06B+1.19C-2.88\text{AB}-1.38\text{AC}-1.25\text{BC}$$ (1)

$$\text{Hexoses yield }\left(\%\right)=6.12+0.09A-0.70B+0.80C-0.20\text{AB}-0.44\text{AC}-0.23\text{BC}$$ (2)

$$\text{HMF yield }\left(\%\right)=0.55+0.36A+0.49B+0.27C+0.30\text{AB}+0.09\text{AC}+0.19\text{BC}$$ (3)

Optimization of the experimental design indicated that, to achieve a high hexose yield of 93%, minimize HMF generation, and maximize sucrose conversion, it is necessary to reduce the acid concentration to 1%, maintain the temperature at 60 °C, and extend the hydrolysis time to up to 120 min. The findings confirm that molasses does not require intensive pretreatment to release simple sugars, thereby enhancing energy efficiency in molasses-based processes by reducing energy-intensive steps. Furthermore, minimizing HMF formation preserves substrate quality, which is essential for preventing microbial inhibition in downstream bioprocesses.

### 3.2 Impact of aeration rates on levulinic acid production

**FIGURE 2:**
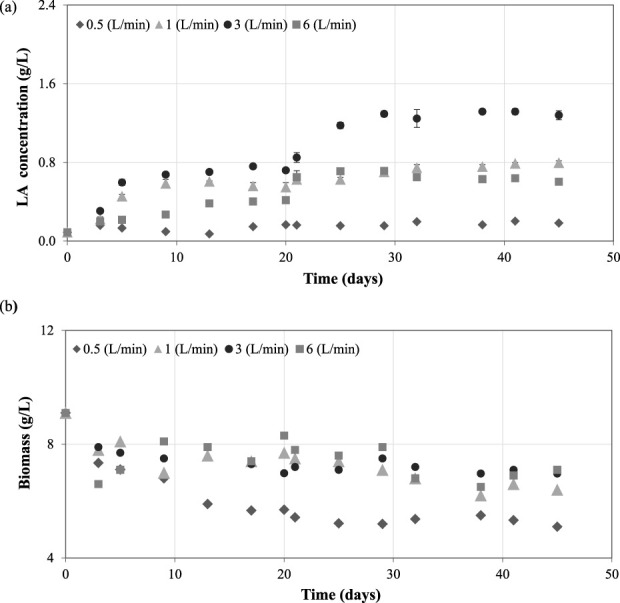
Time-course of levulinic acid (LA) **(a)** and biomass (VS) **(b)** at different aeration rates (0.5–5.0 L/min).

The fermentation of a synthetic hexose mixture using MMC in four SBRs, operated under a feast and famine (F/F) strategy, was conducted for 45 days, with aeration rates ranging from 0.5 to 5.0 L/min. LA was produced under all conditions, with distinct production patterns observed across the evaluated aeration rates (Figure 2a). An initial adaptation phase (10 days) was observed, characterized by a progressive increase in LA concentration and a gradual decline in biomass (VS), followed by a stable operating period with steady levels of both variables (Figures 2a,b). Across all aeration conditions, reducing sugars were efficiently depleted during the feast phase, with removal efficiencies consistently above 95% (Supplementary Figure S2). This behavior has previously been observed in SBR systems subjected to an F/F strategy using xylose as the carbon source (Pinto-Ibieta et al., 2020; Pinto-Ibieta et al., 2023), and similar gradual accumulation has also been reported for other storage polymers, such as PHAs (Huang et al., 2016).

**FIGURE 3:**
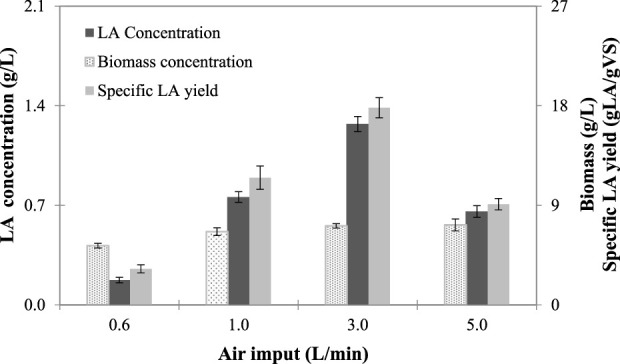
Mean levulinic acid (LA) and biomass concentration in the pseudo-stationary state of the SBRs operated at different aeration rates (0.5–5.0 L/min).

All reactors started at a biomass concentration near 9 g VS/L (Figure 2b); the sharpest decline occurred at 0.5 L/min, reaching an average of 5.4 g VS/L in the pseudo-stationary state, which was significantly lower than 6.6, 7.1 and 7.2 g VS/L at 1.0, 3.0 and 5.0 L/min, respectively, which did not differ significantly among themselves (p > 0.001). The lowest biomass concentration at 0.5 L/min may be associated with oxygen limitation, which can restrict microbial growth by reducing the availability of oxygen as the final electron acceptor and limiting energy generation and substrate assimilation (Vasilakou et al., 2020; Hart and Gorman-Lewis, 2021; Lu et al., 2021; Romo-Buchelly et al., 2022). In contrast, biomass remained stable at 1.0, 3.0, and 5.0 L/min, suggesting that dissolved oxygen (DO) was no longer limiting under these conditions (Romo-Buchelly et al., 2022; Lu et al., 2021). However, excessive oxygenation can increase maintenance demands, reducing biomass yield (Lu et al., 2021; Vasilakou et al., 2020). DO concentrations measured during pseudo-stationary state SBR cycles show distinct profiles across aeration rates, with oxygen-limited conditions at 0.5 L/min, transitional behavior at 1.0 L/min, and predominantly non-limiting DO levels at 3.0 and 5.0 L/min (Supplementary Figure S3). In contrast to the trends observed for biomass concentration, LA concentration, and specific yield differed significantly across aeration rates (p < 0.001): they increased from 0.5 to 1.0 and 3.0 L/min, but decreased at 5.0 L/min. These results suggest that maximum LA production occurs at intermediate aeration rates rather than through linear increases in oxygen supply or biomass concentration. The lowest values were observed at 0.5 L/min (0.30 ± 0.02 g LA/L; 4% ± 1% g LA/g VS). At 1.0 L/min, LA concentration and yield increased to 0.80 ± 0.04 g LA/L and 12% ± 1% g LA/g VS, reaching a maximum at 3.0 L/min (1.30 ± 0.05 g LA/L; 18% ± 1% g LA/g VS) (Figure 3). At 5.0 L/min, both LA concentration and specific yield decreased to about half of those at 3.0 L/min, without a proportional increase in biomass.

LA formation did not increase proportionally with biomass across aeration conditions, consistent with the DO profiles observed during pseudo-stationary state operation, which showed stable intermediate DO levels at aeration rates associated with maximum LA production (Supplementary Figure S3). Given that the biomass and LA did not scale linearly, the effect of varying the aeration rate may reflect shifts in microbial metabolic responses rather than higher cellular growth alone (Blunt et al., 2018).

A similar pattern has been described in *Pseudomonas putida*, reaching 4.2 g/L LA in a fed-batch by maintaining DO at 3 mg O_2_/L (40% saturation) (Kim et al., 2024), consistent with intermediate oxygenation reported as optimal for LA using MMC (Pinto-Ibieta et al., 2021). The same can be observed in the microorganism *Haloferax mediterranei*, where biomass production is inversely proportional to LA concentration under specific oxygen supply (Priya et al., 2022). Additionally, DO could influence microbial metabolism by redirecting sugar degradation toward extracellular polymeric substances or byproducts (Priya et al., 2022; Zhou et al., 2025), which can occur under different oxygen or nutrient conditions.

Thus, regulating the aeration rate to maintain non-limiting but moderately low DO may represent a practical strategy to favor carbon flux toward LA while limiting the formation of alternative products. Alternatively, direct control of DO concentration could provide a more precise operational strategy, as DO reflects the combined effects of aeration rate, oxygen transfer, and microbial activity. In practice, this could be implemented through feedback regulation of aeration based on online DO monitoring, an approach widely applied in aerobic bioprocesses to influence microbial metabolism and process performance (Blunt et al., 2018; Romo-Buchelly et al., 2022).

### 3.3 Effect of organic loading rate (OLR) on levulinic acid yield

**FIGURE 4:**
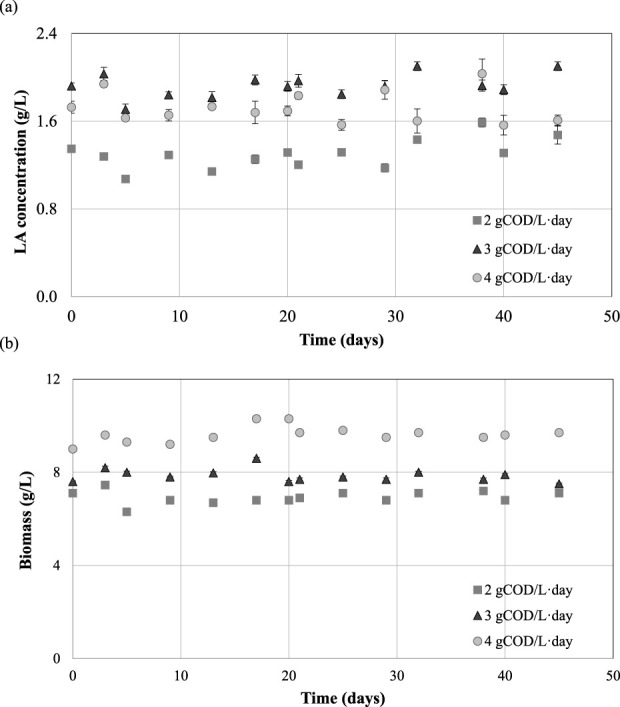
Time-course of LA **(a)** and biomass (VS) **(b)** at different organic loading rates (OLR) (2–4 g COD/L·day).

A second set of experiments was carried out at three OLRs (2, 3, and 4 g COD/(L·day)) for 45 days, setting an aeration rate of 3 L/min, in accordance with the previous results. Unlike aeration, varying the OLR resulted in stable LA and biomass levels throughout the 45 days, with no initial exponential phase (Figures 4a,b). This response may be explained by prior adaptation at 2 g COD/(L·day) and 3 L/min, which may have promoted rapid stabilization and the absence of an initial exponential accumulation phase under all conditions evaluated.

**FIGURE 5:**
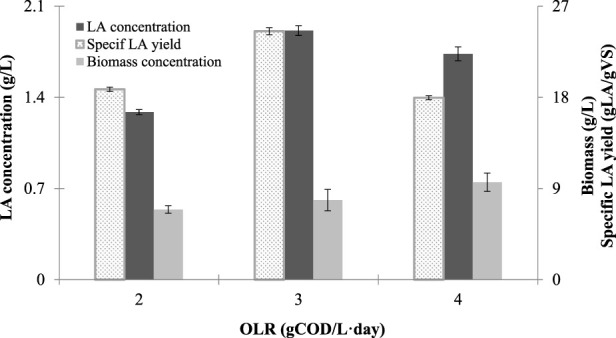
Mean levulinic acid (LA) and biomass concentration in the pseudo-stationary state of the SBRs operated at different organic loading rates (OLR) (2–4 g COD/L·day).

According to the mean LA and biomass concentrations measured at the pseudo-stationary state, SBRs operated at 2, 3, and 4 g COD/(L·day) showed a nonlinear response of LA production to increasing OLR (Figure 5). The highest LA concentration (1.90 ± 0.05 g/L) and specific yield (0.25 ± 0.01 g LA/g VS, corresponding to 25% ± 1%) were obtained at 3 g COD/(L·day). At 4 and 2 g COD/(L·day), LA concentrations were 1.70 ± 0.05 and 1.30 ± 0.06 g/L, respectively, with specific yields of 0.18 ± 0.01 and 0.19 ± 0.01 g LA/g VS (18%–19%). The condition of 3 g COD/(L·day) was significantly higher than those at 2 and 4 g COD/(L·day) (p = 0.012 and p = 0.006, respectively), whereas no significant difference was observed between the latter two conditions (p = 0.810).

The OLR exhibited a direct impact on biomass concentration, demonstrating a progressive increase with OLR, reaching 6.93 ± 0.27, 7.85 ± 0.28, and 9.62 ± 0.35 g SV/L at 2, 3, and 4 g COD/(L·day) (p < 0.001), respectively, and thereby suggesting that the MMC was not inhibited under the evaluated conditions (Campanari et al., 2014). Under the selected aeration rate (3 L/min), the absence of oxygen limitation and the consistently high sugar removal efficiencies (>97%) suggest that the differences observed in LA production across OLR conditions were likely associated with microbial metabolic responses rather than oxygen availability or incomplete substrate utilization (Supplementary Figures S2, 3). In contrast to the findings for biomass generation, LA concentration and yield showed a non-linear trend with an optimum at 3 g COD/(L·day). This divergence indicates an operating range in which LA production was maximized relative to cell growth, highlighting the decoupling between product formation and biomass accumulation under the evaluated conditions. These findings contrast with those of Correa-Galetote et al. (2024), who used a white grape pomace hydrolysate and reported a direct OLR and LA relationship, achieving their highest performance at 4 g COD/(L·day) with 2.45 ± 0.1 g/L of LA, 31% ± 1% g LA/g VS, and 8.0 ± 0.2 g VS/L. Compared with our study using beet molasses at 3 g COD/(L·day), LA concentration and specific yield were 22% and 19% lower, respectively, while biomass was comparable (Table 2). In contrast, relative to the hemicellulosic beet molasses hydrolysate at 2 g COD/(L·day) reported by Pinto-Ibieta et al. (2020), the present research was able to achieve up to a 55% higher LA concentration and 90% higher biomass, with a 17% lower specific yield (Table 2).

**TABLE 2:** Substrate composition and levulinic acid (LA) production by mixed microbial culture using different hydrolysates: comparison with literature reported data.

References	Substrate hydrolysate	OLR (g COD/(L·day))	Sugar composition (g COD/(L·day))	Biomass concentration (g VS/L)	LA concentration (g/L)	LA yield (% g LA/g VS)
Present study	Sugar beet molasses hydrolysate (hexose-rich)	3	Glucose: 1.6 Fructose: 1.4	7.8 ± 0.3	1.9 ± 0.1	25 ± 2
Correa-Galetote et al., 2024	White grape pomace hydrolysate	4	Glucose: 2.6 Xylose: 1.2 Arabinose: 0.2	8.0 ± 0.2	2.5 ± 0.1	31 ± 1
Pinto-Ibieta et al. (2020)	Sugar beet molasses hydrolysate (pentose-rich, inhibitory compounds)	2	Xylose: 1.6 Acetic acid: 0.2 Furfural:0.1 Arabinose:0.1	4.1 ± 0.1	1.2 ± 0.1	30 ± 1

OLR, organic loading rate; COD, chemical oxygen demand; VS, volatile solids.

Taken together, these comparisons indicate that the carbon source shifts the OLR range that maximizes LA formation and redefines the balance between product and growth. To facilitate comparison with previous studies, Table 2 summarizes the hydrolysate compositions and process performance reported in the literature, as well as those obtained in the present work. This comparison suggests that both the dominant sugar type and the presence of microbial growth-inhibiting compounds may influence MMC performance for LA production. In the grape hydrolysate (balanced hexoses-pentoses), the profile coincided with the highest LA concentration and specific yield (2.45 g/L, 31%), with biomass around 8.0 g VS/L. By contrast, the pentose-rich beet-molasses hydrolysate (with inhibitors) was associated with lower LA concentration and biomass (1.2 g/L, 4.1 g VS/L), despite a comparable specific yield (30%). Finally, the hexose-only sugarcane bagasse hydrolysate used in this study showed intermediate LA concentration (1.9 g/L) and biomass comparable to the grape hydrolysate (7.8 vs. 8.0 g VS/L), but the lowest specific yield (25%).

Overall, these trends suggest that MMC performance for LA may be influenced by the combined effects of OLR, sugar composition (hexose–pentose balance), and the presence of inhibitory compounds. In particular, very high pentose fractions (>80%) have been associated with reduced LA accumulation, whereas intermediate levels (30%–35%) have been linked to higher conversion efficiencies in previous studies (Vila-Santa et al., 2021; Kim et al., 2024).

## 4 Conclusion

This study used a process-driven approach to optimize levulinic acid (LA) production from a synthetic hexose mixture that mimics the sugar composition of pretreated beet molasses. The mild hydrolysis conditions (1% v/v H_2_SO_4_, 60 °C, 120 min) yielded a high hexose content with minimal HMF, confirming that molasses does not require severe pretreatment due to its high sucrose content and lack of recalcitrant fibers. In SBRs fed with a synthetic hexose mixture, the selected MMC showed that LA formation was not linearly coupled to oxygen or biomass: 3 L/min aeration rate maximized LA, whereas 0.5 L/min caused oxygen limitation and 5 L/min reduced LA. OLR imposed a non-linear effect with an optimum at 3 g COD/(L·day) (1.90 ± 0.05 g/L; 0.25 ± 0.01 g LA/g VS), while biomass increased with OLR (6.93–9.62 g VS/L), indicating partial decoupling between product formation and growth. Comparisons across sugar composition of reported hydrolysates suggest that balanced hexose-pentose profiles promote higher LA yields, whereas hexose-only feeds enhance biomass at the expense of LA yield. Coupling mild hydrolysis with SBR operation at an aeration rate of 3 L/min and 3 g COD/(L·day) provides an efficient way to produce LA from sugars. Future work should implement DO control, validate inhibitor management using real agro-industrial hydrolysates, assess continuous or semicontinuous operation under pseudo-stationary conditions, and conduct techno-economic and life-cycle analyses.

## Data Availability

The raw data supporting the conclusions of this article will be made available by the authors, without undue reservation.
