# Supplementary material for: SBR operational strategies for directing mixed microbial cultures toward levulinic acid production using hexoses
Source: Front Bioeng Biotechnol. 2026 Jun 1;14:1726739. doi: 10.3389/fbioe.2026.1726739 (PMC13265555; doi:10.3389/fbioe.2026.1726739)
Supplement: Supplementary file 1 [file DataSheet1.docx]

Supplementary Material

**S1. Beet molasses chemical composition**

**Table S1.** Chemical composition of beet molasses after acid hydrolysis

| **Component** | **Content (% w/w)** |
| --- | --- |
| Sucrose | 52.3 |
| Moisture | 13.2 |
| Ashes | 9.0 |
| Crude protein | 8.7 |
| Non-nitrogenous compounds | 8.5 |
| Glucose | 2.8 |
| Fructose | 2.5 |
| Arabinose | 0.7 |
| Levulinic acid | 1.2 |
| Formic acid | 0.6 |
| Acetic acid | 0.6 |

**S2. Reducing sugar concentrations at the beginning and end of SBR cycles**

Reducing sugar concentrations during the SBR operation were monitored to evaluate substrate consumption during each cycle.

**Figure S2.1.** Reducing sugar concentrations at the beginning and end of SBR cycles under different aeration rates.

**Figure S2.2.** Reducing sugar concentrations at the beginning and end of SBR cycles under different organic loading rates.

**S3. Mean dissolved oxygen profiles of SBR cycles under pseudo-stationary state**

Dissolved oxygen profiles were monitored to evaluate oxygen availability during the feast and famine phases.

**Figure S3.** Dissolved oxygen (DO) concentration profiles were monitored throughout the SBR cycles under pseudo-stationary conditions at different aeration rates.

**S4. Experimental Design**

**Table S2.** Experimental design matrix for the acid pre-treatment of beet molasses, varying sulfuric acid concentration (1–10 % v/v), temperature (60–90 °C), and time (30–120 min) in a 3-factor, 2-level design with central points.

| **Run** | **Acid concentration (%)** | **Temperature (°C)** | **Time (min)** |
| --- | --- | --- | --- |
| 1 | 1 | 60 | 30 |
| 17 | 1 | 60 | 30 |
| 21 | 1 | 60 | 30 |
| 36 | 1 | 60 | 30 |
| 6 | 10 | 60 | 30 |
| 27 | 10 | 60 | 30 |
| 28 | 10 | 60 | 30 |
| 34 | 10 | 60 | 30 |
| 4 | 1 | 90 | 30 |
| 19 | 1 | 90 | 30 |
| 20 | 1 | 90 | 30 |
| 32 | 1 | 90 | 30 |
| 16 | 10 | 90 | 30 |
| 18 | 10 | 90 | 30 |
| 25 | 10 | 90 | 30 |
| 29 | 10 | 90 | 30 |
| 2 | 5.5 | 75 | 75 |
| 7 | 5.5 | 75 | 75 |
| 13 | 5.5 | 75 | 75 |
| 23 | 5.5 | 75 | 75 |
| 11 | 1 | 60 | 120 |
| 26 | 1 | 60 | 120 |
| 33 | 1 | 60 | 120 |
| 35 | 1 | 60 | 120 |
| 9 | 10 | 60 | 120 |
| 10 | 10 | 60 | 120 |
| 12 | 10 | 60 | 120 |
| 30 | 10 | 60 | 120 |
| 3 | 1 | 90 | 120 |
| 22 | 1 | 90 | 120 |
| 24 | 1 | 90 | 120 |
| 31 | 1 | 90 | 120 |
| 5 | 10 | 90 | 120 |
| 8 | 10 | 90 | 120 |
| 14 | 10 | 90 | 120 |
| 15 | 10 | 90 | 120 |

**S5. Composition of the culture medium and micronutrient solution used to enrich the synthetic hydrolyzed molasses during the SBR operation**

The culture medium was formulated with the following nutrients per liter: peptone (0.84 g), beef extract (0.44 g), ammonium sulfate (NH₄)₂SO₄ (0.72 g), ammonium chloride (NH₄Cl) (1.1 g), dipotassium phosphate (K₂HPO₄) (0.6 g), monopotassium phosphate (KH₂PO₄) (0.62 g), magnesium sulfate (MgSO₄) (0.42 g), EDTA (0.65 g), calcium chloride (CaCl₂) (0.45 g), and thiourea (0.064 g). Additionally, 8 mL L⁻¹ of a micronutrient solution were added to this medium. The micronutrient solution contained (mg/L): FeCl₃·6H₂O (1500), H₃BO₃ (150), CoCl₂·6H₂O (150), MnCl₂·4H₂O (120), ZnSO₄·7H₂O (120), NaMoO₄·2H₂O (60), CuSO₄·5H₂O (30), and KI (30).

**S6. Statistical model summary for the acid hydrolysis of molasses**

The statistical modeling and experimental optimization of the acid hydrolysis process were performed using Design-Expert® 13. A three-factor, two-level factorial design with center points (3³ runs) was employed to evaluate the effects of acid concentration (A), temperature (B), and reaction time (C) on three response variables: sucrose conversion (%), hexose yield (%), and 5-hydroxymethylfurfural (HMF) yield (%). A two-factor interaction (2FI) model was subsequently fitted to each response, applying distinct transformations according to the characteristics of the datasets. For sucrose conversion, the untransformed 2FI model was statistically significant (p < 0.0001). The hexose yield required a square-root transformation (√y) to achieve significance (p < 0.0001), while the HMF yield was best described by a square-root transformation with λ = 0.34, also yielding a highly significant fit (p < 0.0001). The regression coefficients summary for each response variable is presented in the following tables.

***Table S6.1.*** *Regression coefficients summary for the sucrose conversion model*

| **Factor** | **Coefficient (β)** | **Significance (p < 0.05)** | **Effect direction** |
| --- | --- | --- | --- |
| A – Acid (%) | +3.31 | Significant | Positive |
| B – Temperature (°C) | +3.06 | Significant | Positive |
| C – Time (min) | +1.19 | Significant | Positive |
| AB | −2.88 | Significant | Negative |
| AC | −1.38 | Significant | Negative |
| BC | −1.25 | Significant | Negative |

***Table S6.2.*** *Regression coefficients summary for the hexose yield model*

| **Factor** | **Coefficient (β)** | **Significance (p < 0.05)** | **Effect direction** |
| --- | --- | --- | --- |
| A – Acid (%) | +0.0856 | Not significant | Slight positive |
| B – Temperature (°C) | −0.7012 | Significant | Negative |
| C – Time (min) | +0.7984 | Significant | Positive |
| AB | −0.1985 | Significant | Negative |
| AC | −0.4432 | Significant | Negative |
| BC | −0.2318 | Significant | Negative |

***Table S6.3.*** *Regression coefficients summary for the HMF yield model*

| **Factor** | **Coefficient (β)** | **Significance (p < 0.05)** | **Effect direction** |
| --- | --- | --- | --- |
| A – Acid (%) | +0.3567 | Significant | Positive |
| B – Temperature (°C) | +0.4839 | Significant | Positive |
| C – Time (min) | +0.2708 | Significant | Positive |
| AB | +0.3014 | Significant | Positive |
| AC | +0.0896 | Significant | Slight positive |
| BC | +0.1918 | Significant | Positive |

**S7. Statistical analysis of the effects of aeration rate and organic loading rate (OLR) on LA concentration, biomass formation, and specific LA yield**

**S7. 1** Effect of aeration rate on LA concentration, biomass formation, and specific LA Yield (Data from Section 3.2).

***Table S7.1.*** *LA concentration (g LA/L) across aeration rates: one-way ANOVA and Tukey HSD.*

| **Source of Variation** | **DF** | **SS** | **MS** | **F** | **P** |
| --- | --- | --- | --- | --- | --- |
| Between Groups | 3 | 1.823 | 0.608 | 386.504 | <0.001 |
| Residual | 8 | 0.0126 | 0.00157 |  |  |
| Total | 11 | 1.836 |  |  |  |

| Comparison (L/min) | Diff of Means | p | q | P | P<0.050 |
| --- | --- | --- | --- | --- | --- |
| 3.0 vs 0.6 | 1.098 | 4 | 47.940 | <0.001 | Yes |
| 3.0 vs 5.0 | 0.615 | 4 | 26.864 | <0.001 | Yes |
| 3.0 vs 1.0 | 0.513 | 4 | 22.409 | <0.001 | Yes |
| 1.0 vs 0.6 | 0.585 | 4 | 25.532 | <0.001 | Yes |
| 1.0 vs 5.0 | 0.102 | 4 | 4.456 | 0.054 | No |
| 5.0 vs 0.6 | 0.482 | 4 | 21.076 | <0.001 | Yes |

***Table S7.2.*** *Biomass concentration (g VS/L) across aeration rates: one-way ANOVA and Tukey HSD.*

| **Source of Variation** | **DF** | **SS** | **MS** | **F** | **P** |
| --- | --- | --- | --- | --- | --- |
| Between Groups | 3 | 6.700 | 2.233 | 18.014 | <0.001 |
| Residual | 8 | 0.992 | 0.124 |  |  |
| Total | 11 | 7.692 |  |  |  |

| **Comparison (L/min)** | **Diff of Means** | **p** | **q** | **P** | **P<0.050** |
| --- | --- | --- | --- | --- | --- |
| 5.0 vs 0.6 | 1.872 | 4 | 9.210 | 0.001 | Yes |
| 5.0 vs 1.0 | 0.609 | 4 | 2.994 | 0.227 | No |
| 5.0 vs 3.0 | 0.0886 | 4 | 0.436 | 0.989 | Do Not Test |
| 3.0 vs 0.6 | 1.784 | 4 | 8.775 | 0.001 | Yes |
| 3.0 vs 1.0 | 0.520 | 4 | 2.558 | 0.336 | Do Not Test |
| 1.0 vs 0.6 | 1.264 | 4 | 6.217 | 0.010 | Yes |

***Table S7.3****. Specific LA yield (g LA/g VS) across aeration rates: one-way ANOVA and Tukey HSD.*

| **Source of Variation** | **DF** | **SS** | **MS** | **F** | **P** |
| --- | --- | --- | --- | --- | --- |
| Between Groups | 3 | 327.037 | 109.012 | 187.135 | <0.001 |
| Residual | 8 | 4.660 | 0.583 |  |  |
| Total | 11 | 331.697 |  |  |  |

| **Comparison (L/min)** | **Diff of Means** | **p** | **q** | **P** | **P<0.050** |
| --- | --- | --- | --- | --- | --- |
| 3.0 vs 0.6 | 14.566 | 4 | 33.055 | <0.001 | Yes |
| 3.0 vs 5.0 | 8.718 | 4 | 19.784 | <0.001 | Yes |
| 3.0 vs 1.0 | 6.320 | 4 | 14.343 | <0.001 | Yes |
| 1.0 vs 0.6 | 8.246 | 4 | 18.712 | <0.001 | Yes |
| 1.0 vs 5.0 | 2.398 | 4 | 5.441 | 0.021 | Yes |
| 5.0 vs 0.6 | 5.848 | 4 | 13.271 | <0.001 | Yes |

S7. 2. Effect of organic loading rate (OLR) on LA concentration, biomass, and specific LA Yield (data from Section 3.3)

***Table S7.4****. LA concentration (g LA/L) across OLR: one-way ANOVA and Tukey HSD*

| **Source of Variation** | **DF** | **SS** | **MS** | **F** | **P** |
| --- | --- | --- | --- | --- | --- |
| Between Groups | 2 | 0.624 | 0.312 | 541.345 | <0.001 |
| Residual | 6 | 0.00346 | 0.000576 |  |  |
| Total | 8 | 0.627 |  |  |  |

| **Comparison (g COD/L·d)** | **Diff of Means** | **p** | **q** | **P** | **P<0.050** |
| --- | --- | --- | --- | --- | --- |
| 3 vs 2 | 0.626 | 3 | 45.160 | <0.001 | Yes |
| 3 vs 4 | 0.178 | 3 | 12.860 | <0.001 | Yes |
| 4 vs 2 | 0.448 | 3 | 32.300 | <0.001 | Yes |

***Table S7.5.*** *Biomass concentration (g VS/L) across OLR: one-way ANOVA and Tukey HSD.*

| **Source of Variation** | **DF** | **SS** | **MS** | **F** | **P** |
| --- | --- | --- | --- | --- | --- |
| Between Groups | 2 | 11.239 | 5.619 | 58.491 | <0.001 |
| Residual | 6 | 0.576 | 0.0961 |  |  |
| Total | 8 | 11.815 |  |  |  |

| **Comparison (g COD/L·d)** | **Diff of Means** | **p** | **q** | **P** | **P<0.050** |
| --- | --- | --- | --- | --- | --- |
| 4 vs 2 | 2.694 | 3 | 15.053 | <0.001 | Yes |
| 4 vs 3 | 1.768 | 3 | 9.878 | 0.001 | Yes |
| 3 vs 2 | 0.926 | 3 | 5.176 | 0.025 | Yes |

***Table S7.6.*** *Specific LA yield (g LA/g VS) across OLR: one-way ANOVA and Tukey HSD.*

| **Source of Variation** | **DF** | **SS** | **MS** | **F** | **P** |
| --- | --- | --- | --- | --- | --- |
| Between Groups | 2 | 76.650 | 38.325 | 14.660 | 0.005 |
| Residual | 6 | 15.685 | 2.614 |  |  |
| Total | 8 | 92.335 |  |  |  |

| **Comparison (g COD/L·d)** | **Diff of Means** | **p** | **q** | **P** | **P<0.050** |
| --- | --- | --- | --- | --- | --- |
| 3 vs 4 | 6.564 | 3 | 7.032 | 0.006 | Yes |
| 3 vs 2 | 5.733 | 3 | 6.142 | 0.012 | Yes |
| 2 vs 4 | 0.831 | 3 | 0.890 | 0.810 | No |
